# Prevalence and risk factors for kidney disease among hospitalized PLWH in China

**DOI:** 10.1186/s12981-023-00546-8

**Published:** 2023-07-15

**Authors:** Naxin Zhao, Pan Xiang, Zhili Zeng, Hongyuan Liang, Fang Wang, Jiang Xiao, Di Yang, Sa Wang, Meiling Chen, Guiju Gao

**Affiliations:** 1 Department of Nephrology, Beijing Ditan Hospital, Capital Medical University, Beijing, China; 2 Clinical and Research Center of Infectious Diseases, Beijing Ditan Hospital, Capital Medical University, Beijing, 100015 China; 3 Department of Medical Records and Statistics, Beijing Ditan Hospital, Capital Medical University, Beijing, China

**Keywords:** HIV, AIDS, Kidney disease, Kidney damage, Renal impairment

## Abstract

**Background:**

Kidney disease is an important comorbidity in people living with HIV(PLWH), and is associated with poor outcomes. However, data on renal function of PLWH are limited in China so far. In this study we assessed the prevalence of kidney disease in patients either on antiretroviral therapy (ART) or not respectively in a single center in China and explored the possible risk factors associated.

**Methods:**

In the cross-sectional study, we recruited hospitalized adult PLWH. Demographic characteristics, clinical information and laboratory variables were collected. Kidney disease was defined as estimated glomerular filtration rate (eGFR) < 60 mL/min/1.73 m^2^, and/or isolated hematuria, proteinuria, microalbuminuria. We calculated the prevalence of kidney disease and used logistic regression to assess its associated risk factors.

**Results:**

A total of 501 adult PLWH were enrolled, 446 (89.0%) males and 55 (11.0%) females. The median age was 39 (IQR 30–50) years old. The prevalence of kidney disease was 19.0%, 22 (4.4%) patients with eGFR < 60 mL/min/1.73 m^2^, 53 (10.6%) patients with hematuria, 11 (2.2%) patients with proteinuria, and 40 (8.0%) patients with microalbuminuria. 297 (59.3%) patients were receiving ART. The patients on ART had a higher prevalence of renal disease than those had not been administrated with ART (22.6% vs. 13.7%, P = 0.013). On the multivariate logistic regression analysis among patients not on ART, lower haemoglobin (OR 0.994, 95%CI: 0.902–0.988, *P* = 0.013) were significantly associated with kidney disease. While among those on ART, older age (OR 1.034, 95%CI: 1.003–1.066, *P* = 0.032), lower haemoglobin (OR 0.968, 95%CI: 0.948–0.988, *P* = 0.002) and lower albumin (OR 0.912, 95%CI: 0.834–0.997, *P* = 0.044) were significantly associated with kidney disease.

**Conclusions:**

The prevalence of kidney disease among hospitalized PLWH in China is high, especially in patients on ART. A larger scale study on Chinese outpatient PLWH should be conducted, so as to precisely assess prevalence of kidney disease in general Chinese PLWH.

## Background

Kidney disease is an important comorbidity in people living with HIV (PLWH) and is associated with poor outcomes [1–4]. Lene Ryom et al. [5] reported kidney dysfunction leads to increased morbidity and mortality in PLWH. The prevalence of renal dysfunction in PLWH varies from 11.8 to 76.6% depending on different ethnicities and definition of kidney disease [6, 7]. A study from China [8] reported that 5.8% patients had their estimated glomerular filtration rate (eGFR) below 60 mL/min/1.73 m^2^ after a median of 54 months of ART, but in their study urine tests were not performed. Ying Cao et al. [9] found the prevalence of chronic kidney disease (CKD) in Chinese PLWH who had not initiated ART was 16.1%.

Previous studies indicated that contributing factors to kidney disease in PLWH include older age, hypertension, diabetes mellitus, co-infection with hepatitis B virus (HBV) and hepatitis C virus (HCV) infections, poor virological control, low CD4 count and nephrotoxic drugs [10, 11]. Tenofovir disoproxil fumarate (TDF) is widely used as a first-line antiretroviral agent against HIV which has been proved to be associated with kidney disease [12, 13].

However, data on renal function among PLWH are limitedly reported in China. The aim of our study was to assess the prevalence of kidney disease among PLWH in China involving those either on ART or not and to identify its associated risk factors.

## Methods

### Study design and participants

We conducted a cross-sectional study on PLWH. The patients were all with Chinese nationalities. PLWH above 18 years old who were admitted to Department of Infectious Diseases, Beijing Ditan Hospital, Capital Medical University from February 2019 to January 2020 were observed consecutively. Exclusion criteria as follows: (1) Pregnant women, participants who were suffering from urinary tract infection, patients who had urinary tract malignancy, renal tuberculosis, kidney stones or other obstructive nephropathy; (2) The patients who had missing data which were required to describe kidney damage.

This study was approved by the Ethics Committee of Beijing Ditan Hospital, Capital Medical University. The approval number was Jdlkz 2019-056-02. We obtained informed consent from each participant.

### Data collection and definitions

Demographic characteristics, clinical information and laboratory variables were collected from electronic medical records, including the following parameters: sex, age, weight, height, comorbidities including hypertension, diabetes mellitus, HBV co-infection, HCV co-infection, Syphilis, or tumor, duration of HIV infection, antiretroviral regimen and duration, plasma HIV viral load, CD4 + T-cell count, serum creatinine, urine protein, urine microalbumin, serum concentration of hemoglobin (Hb), albumin (Alb), uric acid (UA), homocysteine (Hcy), procalcitonin (PCT), C reactive protein (CRP), triglycerides (TG), total cholesterol (CHOL), high density lipoprotein cholesterol (HDLC) and low density lipoprotein cholesterol (LDLC). Body-mass index (BMI) was calculated (kg/m²). All laboratory tests were conducted at Clinical Laboratory Department in Beijing Ditan Hospital, Capital Medical University. Serum creatinine was measured using enzymatic assay. The eGFR was calculated by Chronic Kidney Disease Epidemiology Collaboration (CKD-EPI) creatinine equation [14].

Comorbidities, such as hypertension, diabetes, HBV co-infection and HCV co-infection were collected from “past history” in medical records. HBV/HIV co-infection means PLWH with a positive hepatitis B antigen, with a HBV history. HCV/HIV co-infection means those with a positive hepatitis C antibody, with a HCV history. So it contains both chronic HCV infection and prior exposure to HCV. Syphilis refers to patients who had syphilis in their medical history and were positive for Treponema pallidum particle agglutination (TPPA) test or denied history of syphilis, but both toluidine red unheated serum test (TRUST) and TPPA test were positive.

Presence of ≥ + 1 protein or ≥ + 1 occult blood on a spot urine specimen was defined as abnormality on urine dipstick. To minimize the influence of urine sample contamination, patients with abnormal urinalysis were asked to repeat the test. Those with confirmed ≥ + 1 protein or ≥ + 1 occult blood were classified as having proteinuria and haematuria, respectively. Urine microalbumin was measured using immunoturbidimetry with normal reference range < 1.9 mg/dL. Patients who presented with urine microalbumin ≥ 1.9 mg/dL for twice consecutively were classified as having microalbuminuria.

Patients with at least one of the following disorders were considered to have kidney disease: eGFR < 60 mL/min/1.73 m^2^, hematuria, proteinuria, or microalbuminuria.

### Statistical analysis

All analyses were performed using Statistical Package for Social Sciences (SPSS) 20.0 software (SPSS Institute, Chicago IL, USA). We conducted One-Sample Kolmogorov-Smirnov test for homogeneity of quantitative data. We presented data with normal distribution as mean ± standard deviation (SD), including BMI, Alb and LDLC. Data without a normal distribution was presented as median (interquartile range), including age, duration of HIV, antiretroviral duration, plasma HIV viral load, CD4 T-cell count, serum creatinine, Hb, UA, Hcy, PCT, CRP, TG, CHOL and HDLC. Categorical variables were presented as frequency and percentage. Chi-squared test was used to compare prevalence of kidney disease in patients on ART and those not on ART. Univariate and multivariate logistic regression analyses were carried out to estimate associations between potential risk factors and the development of kidney disease. A two-tailed P values < 0.05 were considered statistically significant.

## Results

**Fig. 1:**
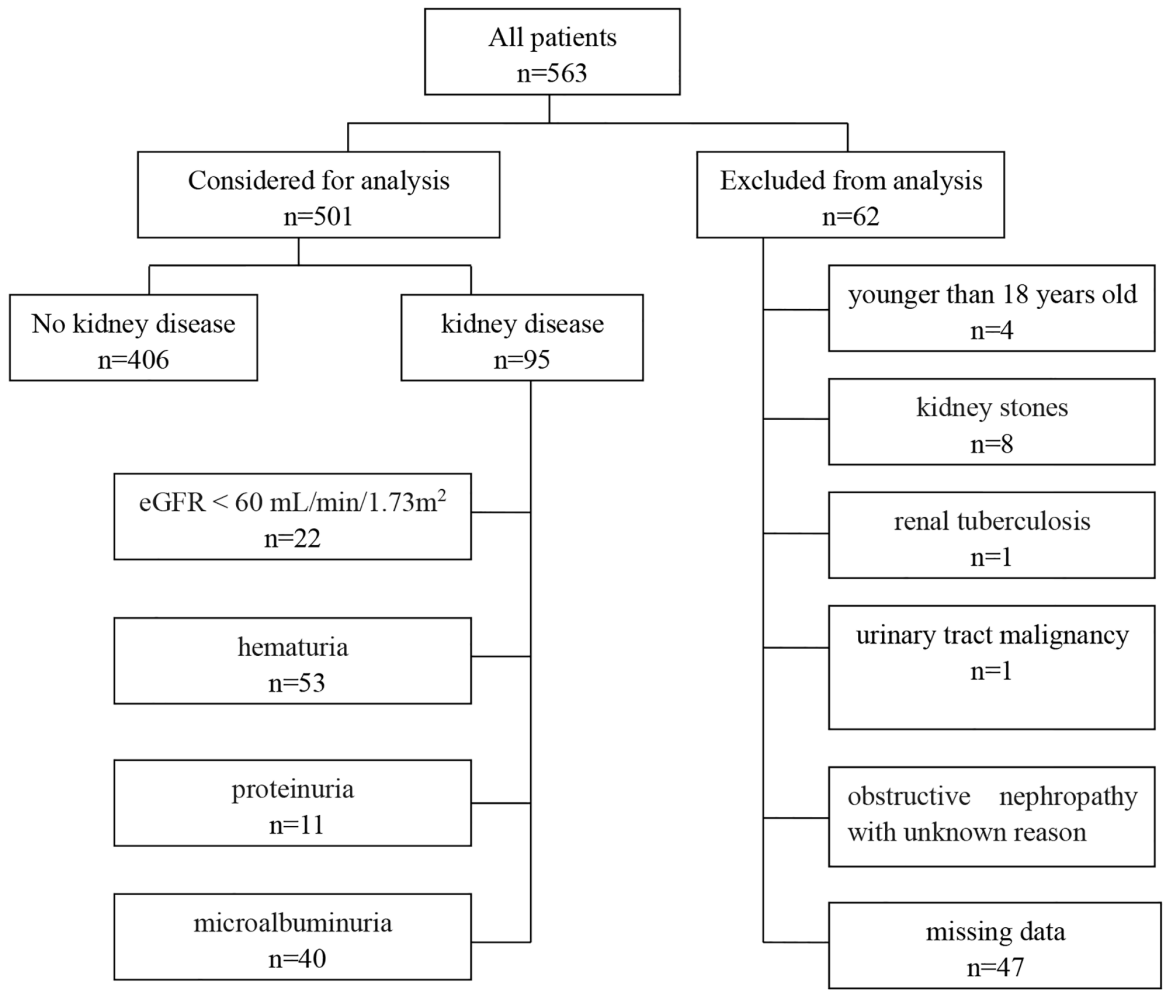
Study flow chart detailing all the inclusion and exclusion of patients. eGFR: estimated glomerular filtration rate

A total of 563 PLWH were admitted to Department of Infectious Diseases, Beijing Ditan Hospital, Capital Medical University during the study period. Forty seven individuals with eGFR ≥ 60 mL/min/1.73 m^2^ had missing data for urine test, leading to impossibility to identify whether they had kidney disease. Finally, 501 (88.99%) Chinese adult PLWH were enrolled (Fig. 1).

### Patient characteristics

There were 446 (89.0%) males and 55 (11.0%) females. Median age was 39 (IQR 30–50) years old, and the youngest patient was 19 years old, while the oldest one was 83 years old. BMI was 21.65 ± 3.57 kg/m^2^. HBV, HCV and Syphilis diagnoses were establised in 33 (6.6%), 16 (3.2%), and 139 (27.7%) patients, respectively. 40 (8%), 39 (7.8%), and 37 (7.4%) patients had hypertension, diabetes, and tumor, respectively. Median duration of HIV was 8 (IQR 0–57.5) months.

The longest duration of HIV was 276 months. 153 (30.5%) patients were newly diagnosed with HIV infection (diagnosed within 1 month). 297 (59.3%) patients were receiving antiretroviral therapy, of whom 239 (47.7%) patients were receiving TDF-containing regimen. The majority of these patients (165, 32.9%) were prescribed ART with a combination of TDF, lamivudine (3TC), and efavirenz (EFV). 65(13.0%) patients were receiving protease inhibitors (PIs)-containing regimen. 64(12.8%) patients were prescribed with lopinavir/ritonavir and one patient with boosted darunavir. None of the patients were prescribed with atazanavir. 46(9.2%) patients were administered with TDF in combination with PIs. In the patients on ART, median duration of ART was 12 (IQR 2–47) months. The longest duration of ART was 216 months. 161 (54.2%) patients had HIV viral load < 20 copies/mL or undetectable. Median CD4 + T cell count was 215 (IQR 66–458) cells/µL, with 143 (48.1%) patients presenting with a CD4 + T cell count < 200 cells/µL. More information is available in Table 1.

**Table 1:** Demographic and clinical characteristics of participants

	Total n = 501	Not on ART n = 204(40.7%)	On ART n = 297(59.3%)
Age, median (IQR), y	39 (IQR 30–50)	37 (IQR 28–46)	40 (IQR 31–52)
sex, No. (%)			
Male	446(89.0%)	193(94.6%)	253(85.2%)
Female	55(11.0%)	11(5.4%)	44(14.8%)
BMI, Mean ± SD, kg/m^2^	21.65 ± 3.57	21.29 ± 3.59	21.91 ± 3.54
HBV coinfection, No. (%)	33(6.6%)	12(5.9%)	21(7.1%)
HCV coinfection, No. (%)	16(3.2%)	0(0.0%)	16(5.4%)
Syphilis, No. (%)	139(27.7%)	58(28.4%)	81(27.3%)
Hypertension, No. (%)	40(8%)	12(5.9%)	28(9.4%)
Diabetes mellitus, No. (%)	39(7.8%)	12(5.9%)	27(9.1%)
Tumor, No. (%)	37(7.4%)	9(4.4%)	28(9.4%)
duration of HIV, Median (IQR), mo	8 (IQR 0–57.5)	0 (IQR 0–1)	24 (IQR 6.5–84)
HIV viral load, median (IQR), copies/mL	2239 (IQR0–167,717)	195,721(IQR80916–505,206)	1(IQR 0–408)
CD4 count, Median (IQR), cells/uL	106 (IQR 28.5–362)	39 (IQR11–149)	215(IQR 66–458)
< 200 cells/ uL, No. (%)	308(61.5%)	165(80.9%)	143(48.1%)
≥ 200 cells/ uL, No. (%)	198(38.5%)	39(19.1%)	154(51.9%)
Kidney damage, No. (%)	95(19.0%)	28(13.7%)	67(22.6%)
eGFR, median (IQR), mL/min/1.73 m^2^	115 (103–124)	116 (105–125)	105 (76–119)

IQR: interquartile range; BMI: Body-mass index; SD: standard deviation; HBV: hepatitis B virus; HCV: hepatitis C virus; HIV: human immunodeficiency virus; ART: antiretroviral treatment; TDF: tenofovir disoproxil fumarate; eGFR: estimated glomerular filtration rate

### Prevalence of renal dysfunction

Of the total study population, 95 patients had kidney disease. The prevalence of kidney disease was 19.0%. 22 (4.4%) patients had eGFR < 60 mL/min/1.73 m^2^, 53 (10.6%) patients had hematuria, 11 (2.2%) patients had proteinuria, and 40 (8.0%) patients had microalbuminuria. Some patients had two or more disorders simultaneously, so the total number is not equal to 95. The patients on ART had a higher prevalence of renal disease than those not on ART(22.6% vs. 13.7%, P = 0.013).

### Association between renal function and Potential risk factors

Table 2 shows the strength of associations between kidney disease and its potential risk factors in PLWH not on ART. Using univariate analysis, kidney disease was found to be significantly associated with older age, hypertension, diabetes mellitus, and CD4 + T cell count. There was no significant association between kidney disease and sex, BMI, HBV coinfection, Syphilis, tumor, duration of HIV, HIV viral load, hemoglobin, albumin, UA, CRP, PCT, HCY, HDLC or LDLC. But in the multivariate logistic regression analysis, only lower haemoglobin (OR 0.994, 95%CI: 0.902–0.988, *P* = 0.013) were significantly associated with kidney disease.

**Table 2:** Logistic Regression Analysis for Identification of Predictors of kideny disease in PLWH not on ART

	Univariate Model Unadjusted OR(95%CI)	P	Multivariate Model Adjusted OR(95%CI)	P
**Age**	**1.058(1.020–1.097)**	**0.003**	1.046(0.985–1.111)	0.142
Female	0.000(0.000-)	0.999	0.000(0.000-)	0.998
BMI	1.105(0.991–1.232)	0.072	1.099(0.920–1.312)	0.298
HBV coinfection	0.556(0.069–4.479)	0.581	1.046(0.072–15.304)	0.974
Syphilis	1.481(0.639–3.436)	0.360	2.520(0.685–9.267)	0.164
**Hypertension**	**11.400(3.319–39.154)**	**< 0.001**	6.210(0.771–50.038)	0.086
**Diabetes mellitus**	**5.248(1.538–17.912)**	**0.008**	5.030(0.512–49.392)	1.166
Tumor	1.857(0.366–9.429)	0.455	1.825(0.147–22.666)	0.640
duration of HIV	0.996(0.979–1.014)	0.666	0.997(0.975–1.020)	0.812
HIV viral load	1.000(1.000–1.000)	0.456	1.000(1.000–1.000)	0.578
**CD4 count**	**1.003(1.001–1.005)**	**0.013**	1.008(1.000-1.015)	0.057
>200	1.500(0.588–3.829)	0.396	0.870(0.368–2.059)	0.109
**Hb**	0.986(0.969–1.003)	0.101	**0.944(0.902–0.988)**	**0.013**
PCT	1.049(0.984–1.119)	0.140	1.055(0.975–1.141)	0.181
UA	1.003(1.000-1.006)	0.057	0.997(0.992–1.003)	0.361
ALB	1.026(0.953–1.104)	0.498	1.151(0.941–1.406)	1.171
HCY	0.993(0.973–1.045)	0.777	0.958(0.849–1.081)	0.484
CRP	1.002(0.990–1.013)	0.779	0.995(0.976–1.014)	0.614
CHOL	1.546(0.959–2.493)	0.074	4.579(0.177-118.241)	0.359
TG	1.195(0.939–1.521)	0.147	0.644(0.200-2.076)	0.461
HDLC	3.733(0.980-14.219)	0.054	0.797(0.011–60.412)	0.918
LDLC	1.029(0.568–1.866)	0.924	0.212(0.008–5.959)	0.362

CI: confidence interval; OR: odds ratio; Hb: hemoglobin; UA: uric acid; ALB: albumin; Hcy: homocysteine; PCT: procalcitonin; CRP: C reactive protein; CHOL: total cholesterol; TG: triglycerides; HDLC: high density lipoprotein cholesterol; LDLC: low density lipoprotein cholesterol; see Table 1. The P values in bold are statistically significant P values

Table 3 shows the strength of associations between kidney disease and its potential risk factors in PLWH on ART. Using univariate analysis, kidney disease was found to be significantly associated with older age, hypertension, diabetes mellitus, duration of ART, lower hemoglobin and albumin, and higher PCT and CRP. There was no significant association between kidney disease and sex, BMI, HBV coinfection, HCV coinfection, Syphilis, tumor, duration of HIV, HIV viral load, CD4 + T cell count, UA, HCY, HDLC or LDLC. In the multivariate logistic regression analysis, older age (OR 1.034, 95%CI: 1.003–1.066, *P* = 0.032), lower haemoglobin (OR 0.968, 95%CI: 0.948–0.988, *P* = 0.002) and lower albumin(OR 0.912, 95%CI: 0.834–0.997, *P* = 0.044) were significantly associated with kidney disease.

**Table 3:** Logistic Regression Analysis for Identification of Predictors of kidney disease in PLWH on ART

	Univariate Model Unadjusted OR(95%CI)	P	Multivariate Model Adjusted OR(95%CI)	P
**Age**	**1.048(1.026–1.071)**	**< 0.001**	**1.034(1.003–1.066)**	**0.032**
Female	1.545(0.757–3.156)	0.232	0.754(0.256–2.217)	0.608
BMI	1.010(0.933–1.093)	0.805	1.077(0.965–1.203)	0.185
HBV coinfection	0.552(0.158–1.934)	0.353	0.567(0.120–2.691)	0.475
HCV coinfection	1.606(0.538–4.795)	0.396	0.638(0.136–2.985)	0.568
Syphilis	0.574(0.294–1.119)	0.103	0.394(0.146–1.060)	0.065
**Hypertension**	**3.451(1.550–7.682)**	**0.002**	1.004(0.276–3.649)	0.995
**Diabetes mellitus**	**5.240(2.315–11.864)**	**< 0.001**	3.271(0.921–11.615)	0.067
Tumor	0.930(0.361–2.397)	0.880	0.600(0.170–2.109)	0.425
duration of HIV	1.004(1.000-1.009)	0.056	1.000(0.991–1.009)	0.923
**duration of ART**	**1.009(1.003–1.016)**	**0.007**	1.015(1.000-1.030)	0.051
HIV viral load	1.000(1.000–1.000)	0.705	1.000(1.000–1.000)	0.801
≥ 20 copies/mL	0.690(0.394–1.211)	0.196	0.749(0.299–1.879)	0.538
CD4 count	1.000(0.999–1.001)	0.769	1.000(0.999–1.001)	0.945
>200 cells/ uL	1.102(0.639–1.902)	0.726	1.239(0.408–3.761)	0.706
TDF-containing regimen	1.404(0.730–2.700)	0.309	0.468(0.169–1.296)	0.144
**Hb**	**0.977(0.967–0.988)**	**< 0.001**	**0.968(0.948–0.988)**	**0.002**
**PCT**	**1.198(1.002–1.434)**	**0.048**	1.127(0.919–1.383)	0.251
UA	1.001(0.999–1.003)	0.196	1.002(1.000-1.004)	0.081
**ALB**	**0.900(0.857–0.946)**	**< 0.001**	**0.912(0.834–0.997)**	**0.044**
HCY	0.993(0.969–1.018)	0.593	0.990(0.958–1.023)	0.542
**CRP**	**1.006(1.001–1.011)**	**0.018**	1.003(0.994–1.011)	0.567
CHOL	1.175(0.943–1.463)	0.151	1.633(0.523–5.094)	0.398
TG	1.076(0.963–1.202)	0.193	1.001(0.727–1.378)	0.995
HDLC	1.025(0.421–2.495)	0.957	2.496(0.400-15.590)	0.328
LDLC	1.019(0.727–1.430)	0.913	0.530(0.150–1.882)	0.326

TDF: tenofovir disoproxil fumarate; see Tables 1 and 2. The P values in bold are statistically significant P values.

## Discussion

In this study, we assessed the prevalence of kidney disease in Chinese adult PLWH and assessed the associated risk factors of kidney disease in this population. Similar to previous studies [9], our study demonstrated that the prevalence of kidney disease among Chinese adult PLWH was 19.0%. Furthermore, our present study showed the prevalence of kidney disease was 13.7% among patients not on ART and 22.6% among those on ART. Kedar Joshi et al. [12] discovered a decrease in renal function in PLWH in Asia with long-term antiretroviral therapy, which is in line with our study.

Majority of the patients on ART received TDF-containing regimen, which is one of the possible reasons why the prevalence of kidney disease among patients on ART was higher than those not on. Prior studies [15–18] confirmed that TDF was associated with kidney disease. The influence of TDF on kidney function is a well-established phenomenon with specific pathogenetic mechanisms.

Ying Cao et al. [9] ascertained that advanced age and higher viral load were significantly correlated with development of CKD. Similar to what they found, we discovered that older ager was significantly correlated with kidney disease. In the multivariate logistic regression analysis, higher viral load was not associated with kidney disease in our study,. The possible reasons are as follows: (1) The duration of HIV infection was short, without making any speculations on the possible damage of HIV on the kidney. (2) Kidney damage might have been drug-related in ART-experienced PLWH. This can also explain why low CD4 cell count is not associated with renal damage in our study, while prior study [11, 15, 19] confirmed low CD4 count was a risk factor for kidney damage.

Eduardo Shahar et al. [13] reported co-infection with HCV is significantly associated with the decline in kidney function among PLWH. Amanda Mocroft et al. [20] also found the odds of CKD were increased in participants with HBV or HCV in PLWH. But there was no significant association between kidney damage and HBV or HCV coinfection in our study. The reason may be that the number of patients with HBV or HCV in our study is relatively small, moreover, in our study HCV co-infection contains both chronic HCV infection and prior exposure to HCV.

Consistent with previous studies [21, 22], lower hemoglobin and lower albumin were associated with kidney disease among patients on ART in our study. Lin Pu et al. [21] reported that patients with severe acute kidney injury (AKI) had lower serum albumin and hemoglobin levels among PLWH. A study in the United States [22] found lower serum albumin levels were strongly associated with kidney function decline in HIV-infected women independent of albuminuria, HIV disease status, and BMI.

Prior studies found that kidney disease is significantly associated with hypertension, diabetes mellitus, duration of HIV, ART, CRP, and CHOL [23–26]. Through all these studies, we can see that the risk factors of kidney damage are various, so it is very important to monitor the renal function and comprehensively manage the risk factors in PLWH. But in the multivariate logistic regression analysis in our study, we did not find kidney disease is significantly associated with those factors. The reason may be that the patients in our study are younger and the duration of HIV is shorter than those patients in prior studies, in addition, our sample size is relatively small.

Our study has following advantages. Firstly, we used enzymatic method to quantify serum creatinine, which is more accurate than Jaffe method [27]. Secondly, this is the first clinical study to assess the prevalence and correlative factors of kidney disease in PLWH both on ART and not in China. We found that patients on ART had a higher prevalence of renal disease than those not on ART. Thirdly, we detected urine microalbumin to identify patients with early renal damage.

However, our study also has limitations. Firstly, we used CKD-EPI creatinine equation to calculate eGFR, which may underestimate the prevalence of renal dysfunction compared to CKD-EPI creatinine-cystatin C equation [28]. Secondly, this is a single-center study, which largely limits its generalizability. And this study was done among patients admitted to hospital. Opportunistic infection was the main reason for their admission, which could have contributed to the transient renal disease. And since this cohort represented hospitalized PLWH, the rate of renal disease that we reported may not reflect that of the general population of PLWH. Thirdly, the sample size is relatively small. A prospective larger scale multi-center study on general population of PLWH to determine the prevalence and potential risk factors responsible for kidney disease in Chinese PLWH should be conducted in the future.

## Conclusion

In conclusion, the prevalence of kidney disease in hospitalized PLWH in China is high, especially in the patients on ART. A larger scale study on Chinese outpatient PLWH should be conducted, so as to precisely assess prevalence of kidney disease in general Chinese PLWH.

## Data Availability

The data are available from the corresponding author with reasonable request.

## Abbreviations

HIV: Human immunodeficiency virus
PLWH: People living with HIV
AIDS: Acquired Immune Deficiency Syndrome
ART: Antiretroviral therapy
eGFR: Estimated glomerular filtration rate
TDF: Tenofovir disoproxil fumarate
3TC: Lamivudine
EFV: Efavirenz
HBV: Hepatitis B virus
HCV: Hepatitis C virus
Hb: Hemoglobin
Alb: Albumin
UA: Uric acid
Hcy: Homocysteine
PCT: Procalcitonin
CRP: C reactive protein
TG: Triglyceride
CHOL: Total cholesterol
HDLC: High density lipoprotein cholesterol
LDLC: Low density lipoprotein cholesterol
BMI: Body-mass index
CKD-EPI: Chronic Kidney Disease Epidemiology Collaboration
CKD: Chronic kidney disease
Scr: Serum creatinine
SPSS: Statistical Package for Social Sciences
SD: Standard deviation
IQR: Interquartile range
CI: Confidence interval
OR: Odds ratio
